# Quantification of allelic differential expression using a simple Fluorescence primer PCR-RFLP-based method

**DOI:** 10.1038/s41598-019-42815-5

**Published:** 2019-04-19

**Authors:** Changzhi Zhao, Shengsong Xie, Hui Wu, Yu Luan, Suqin Hu, Juan Ni, Ruiyi Lin, Shuhong Zhao, Dingxiao Zhang, Xinyun Li

**Affiliations:** 1 Key Laboratory of Agricultural Animal Genetics, Breeding, and Reproduction of the Ministry of Education & Key Lab of Swine Genetics and Breeding of Ministry of Agriculture and Rural Affairs, Huazhong Agricultural University, Wuhan, 430070 P.R. China; 2 The Cooperative Innovation Center for Sustainable Pig Production, Huazhong Agricultural University, Wuhan, 430070 P.R. China

**Keywords:** Genetic engineering, Gene expression profiling

## Abstract

Allelic differential expression (ADE) is common in diploid organisms, and is often the key reason for specific phenotype variations. Thus, ADE detection is important for identification of major genes and causal mutations. To date, sensitive and simple methods to detect ADE are still lacking. In this study, we have developed an accurate, simple, and sensitive method, named fluorescence primer PCR-RFLP quantitative method (fPCR-RFLP), for ADE analysis. This method involves two rounds of PCR amplification using a pair of primers, one of which is double-labeled with an overhang 6-FAM. The two alleles are then separated by RFLP and quantified by fluorescence density. fPCR-RFLP could precisely distinguish ADE cross a range of 1- to 32-fold differences. Using this method, we verified *PLAG1* and *KIT*, two candidate genes related to growth rate and immune response traits of pigs, to be ADE both at different developmental stages and in different tissues. Our data demonstrates that fPCR-RFLP is an accurate and sensitive method for detecting ADE on both DNA and RNA level. Therefore, this powerful tool provides a way to analyze mutations that cause ADE.

## Introduction

In diploid organisms, the expression level of two alleles are equal for most of the genes, whereas some of them are significantly different. This phenomenon is named allelic differential expression (ADE)^1–3^. Nowadays, ADE occurs because of several reasons, including genomic imprinting^4^, X-chromosome inactivation^5^, and mutation of regulatory elements^6^. ADE plays an important role in phenotypic determination^7–10^. Therefore, detection of ADE and associated casual mutations in the regulatory elements is important for uncovering the molecular mechanisms of economic traits of farm animals or diseases of human beings^11^.

Recently, many approaches have been applied to determine ADE, such as high-throughput DNA/RNA sequencing^12–18^, DNA microarray^19–21^, Taqman PCR^22,23^, allele specific real-time PCR^24^, and digital PCR^25^. These methods lead to the detection of major genes associated with human disease, causal mutations in cis-regulatory elements, loss of function alleles, and others^26–29^. Although various bioinformatics tools, including IDP-ASE^30^, cisASE^31^ and QuASAR^32^, have been developed to detect ADE based on high-throughput sequencing data, many factors can still hamper the accuracy of ADE, such as statistical confidence^33^, mapping bias^34,35^, and the initial reference alignment issues^36^. In particular, mapping bias gives rise to a preferential alignment to the reference allele, which creates a major obstacle in ADE analysis^37^. Microarray can be used to detect ADE, but the noise and the quality of the chip may confound the results. In addition, a standard threshold to estimate significant ADE is still lacking in DNA microarray method, which may produce a certain proportion of false positive results. Furthermore, high-throughput methods have some limitations, including false positive and false negative issues, high consumption of time and cost, and that the potential ADE still needs to be validated by PCR. Current PCR-based methods often use two pairs of primers with difference at the 3′-end nucleotide, which may cause bias due to variation in amplification efficiency. Previously, SNaPshot was frequently used to detect ADE due to its low background noise and high sensitivity^38,39^. This method includes a first multiplexed PCR step, followed by a single-base extension reaction by the use of an extension primer with different lengths directly adjacent to a SNP. The one nucleotide extension with distinct fluorescent dideoxynuleotides reflects the SNP allele. Subsequently, alleles were analyzed by capillary electrophoresis and DNA sequencer. By analyses of sequencing data via GeneMapper® software^40,41^, the peaks of two alleles representing ADE are recorded. Consequently, SNaPshot is a method with many steps and requires unusual laboratory equipment (e.g., capillary electrophoresis and DNA sequencer), leading to limited dissemination of this technique. Thus, development of a simple efficient, and cost-effective methods is still needed to detect ADE.

Here we have developed a simple and robust method combining RT-PCR and RFLP for detection of ADE, which is named fluorescence primer PCR-RFLP quantitative method (fPCR-RFLP). This method uses only one pair of primers with one of them is double-labeled with an overhang 6-FAM at its 5′ terminal. Two alleles are then separated by RFLP and agarose electrophoresis, followed by gel imaging analysis with a scanner detecting FAM signals. The ratio of ADE is quantified based on the fluorescent intensity of corresponding bands. Recently, we have resequenced a total of 68 pig genomes of four breeds representing Chinese domestic pigs, Western domestic pigs, Asian wild boars, and Western wild pigs, and identified numerous SNPs based on alignment against Swine reference genome (Sus scrofa 10.2). To illustrate the utility of our method, we validated two ADE genes, *PLAG1* and *KIT*, expressed in different tissues and at different developmental stages. Therefore, we establish fPCR-RFLP as a simple, sensitive and accurate method, which could provide a powerful way to detect mutations that cause ADE in many species.

## Results

### Development of Fluorescence primer PCR-RFLP (fPCR-RFLP) for quantifying ADE

In the fPCR-RFLP method, the first round of PCR with cDNA as template was performed using gene specific primers. The resulting PCR product was then amplified using a fluorescently labeled primer in the second round of PCR. These PCR products were digested with an appropriate restriction enzyme. Subsequently, restriction fragments were separated by agarose gel electrophoresis. The labeled fragments can be analyzed by peak area ratio.

**Figure 1:**
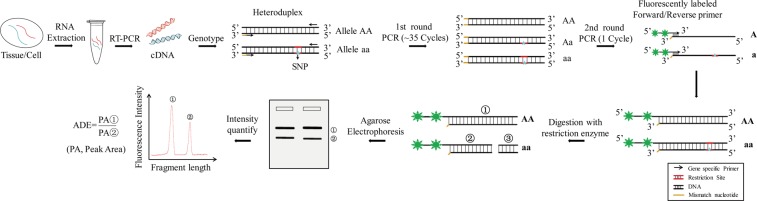
Schematic diagram of Fluorescence PCR-RFLP (fPCR-RFLP) method. Total RNA was extracted from tissues or cells. cDNAs were synthesized by RT-PCR. Heteroduplex was validated by genotyping and then subjected to the first round of PCR (about 35 cycles) using a gene-specific primer. Next, the PCR product was used as template for the second round of PCR with one cycle using one fluorescently labeled primer. This step generated two alleles with over-hang labeling. The labeled PCR products were digested by restriction enzyme. The digested products were separated by agarose gel electrophoresis. Finally, only the fluorescently labeled fragments were detected by a laser detection system. The ratio of peak area represented the ADE.

The detailed workflow of fPCR-RFLP method was illustrated in Fig. 1. The major steps were as follows. To detect ADE, cDNAs were first synthesized by RT-PCR. Second, genotyping was performed to identify heterozygous types. Third, the target fragment of heterozygosity was amplified firstly using universal PCR. There were three types of PCR products, including two kinds of homodimer (AA and aa) and one heterodimer (Aa). Then, the second round of PCR was performed with one cycle using FAM labeled primers and the products of first round PCR as template. Two alleles labeled with FAM (AA-FAM and aa-FAM) were generated after the second round of PCR. Afterward, the two alleles were digested using an appropriate restriction enzyme, followed by gel electrophoresis to detect the AA-FAM and aa-FAM. Fluorescence density of the bands was analyzed using software Fujifilm FLA-5100 Scanner, and the corresponding peak area was considered as the expression level of the two alleles. The ADE was then calculated as the peak area ratio of the two bands.

### The fluorescence density is related to the copy number of DNA strand and the ways of FAM labeling

**Figure 2:**
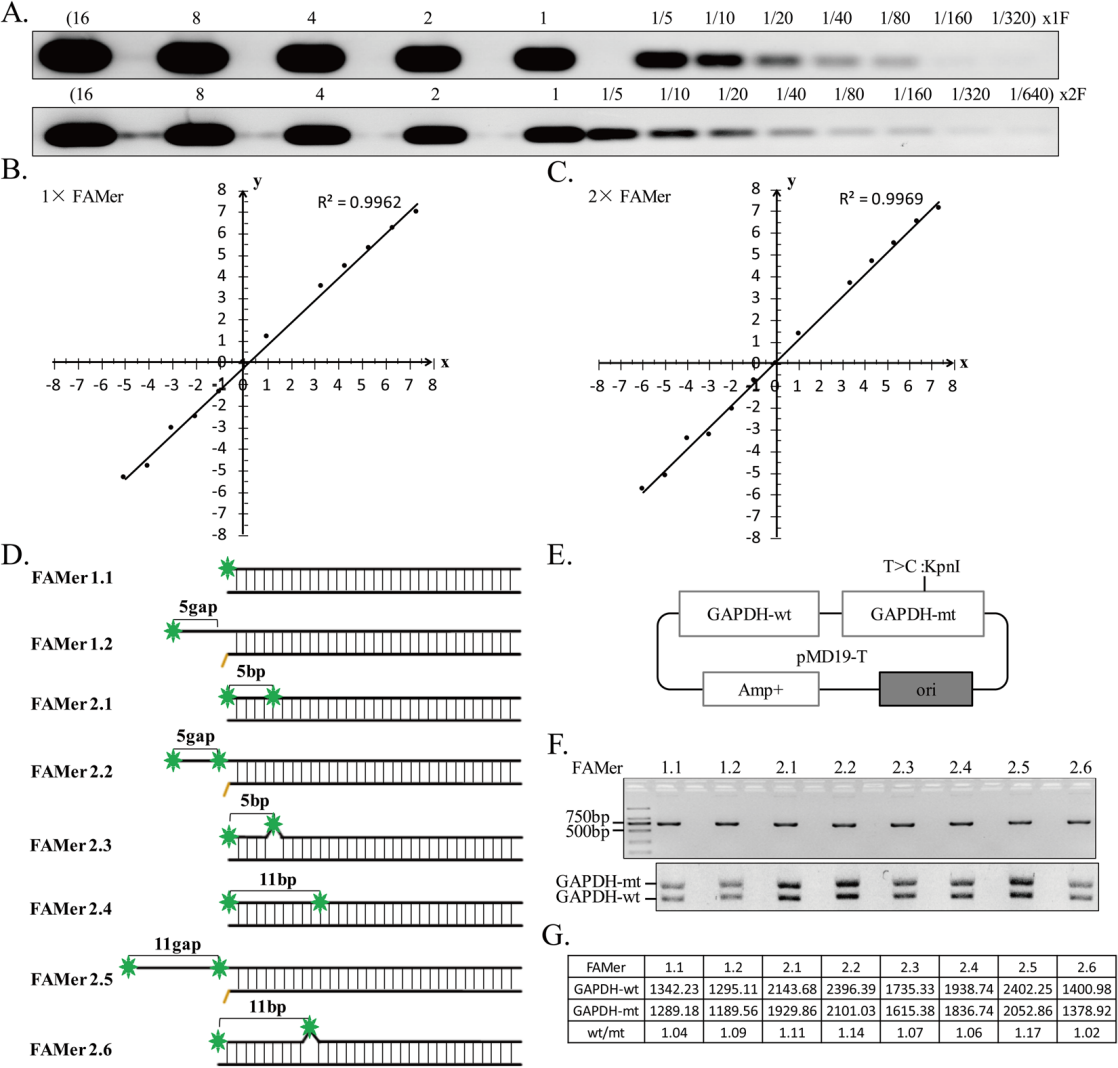
Evaluation of fluorescence density of the PCR products amplified with different FAM labeled primers. (**A**–**C**) Fluorescence density analysis of serial diluted 1× FAMer or 2× FAMer-labeled primers. (**D**) The diagram of PCR products with 8 different types of FAM-labeled primers. Green asterisk represents the 6-FAM-labeled nucleotide. (**E**) A vector containing normal and mutant GAPDH DNA copy was used as PCR template for the fluorescence density analysis. (**F**) The fPCR-RFLP results of 8 different FAM-labeled primers. (**G**) The fluorescence density of wild type (wt) and mutant (mt) GAPDH copies revealed that the ratio of wt/mt was approximately 1.0. Full-length gels are presented in Supplementary Fig. 3.

For the fPCR-RFLP method, the fluorescence density was very important. First, it should be strong enough for detection. Second, it could be representative of the copy number of DNA strand. Therefore, the fluorescence density was analyzed via different ways. First, one FAM (1× FAMer) and two FAM (2× FAMer) labeled primers were serial diluted (from 16× to 1/320× or 1/640×). Serial dilution assay showed a strong correlation between fluorescence density and the copy number of DNA strand (*R*^2^ = 0.9962 for 1× FAMer; *R*^2^ = 0.9969 for 2× FAMer) (Fig. 2A–C). The raw and normalized fluorescence density was shown in Supplementary Table S2. The raw value was normalized using Log_2_. Furthermore, variations in fluorescence density of 8 different types of FAM labeling were tested (Fig. 2D). A plasmid with normal and mutant *GAPDH* genes was used as template (Fig. 2E). The products with two FAM-labeled primer were brighter than those with one FAM-labeled primer. The overhang FAM-labeled primers of FAMer 2.2 and FAMer 2.5 were the brightest two (Fig. 2F,G). FAMer 2.5 had longer overhang, FAMer 2.2 was thus chosen for subsequent experiments. Furthermore, three and five FAM-labeled primers were also tested. The results showed that the fluorescence density was weakened when the space of two FAM was less than 3 nucleotides (see Supplementary Fig. S1). We recommended a 5-bp space between two FAM-labeled nucleotides.

### Assessment of the accuracy of fPCR-RFLP method

**Figure 3:**
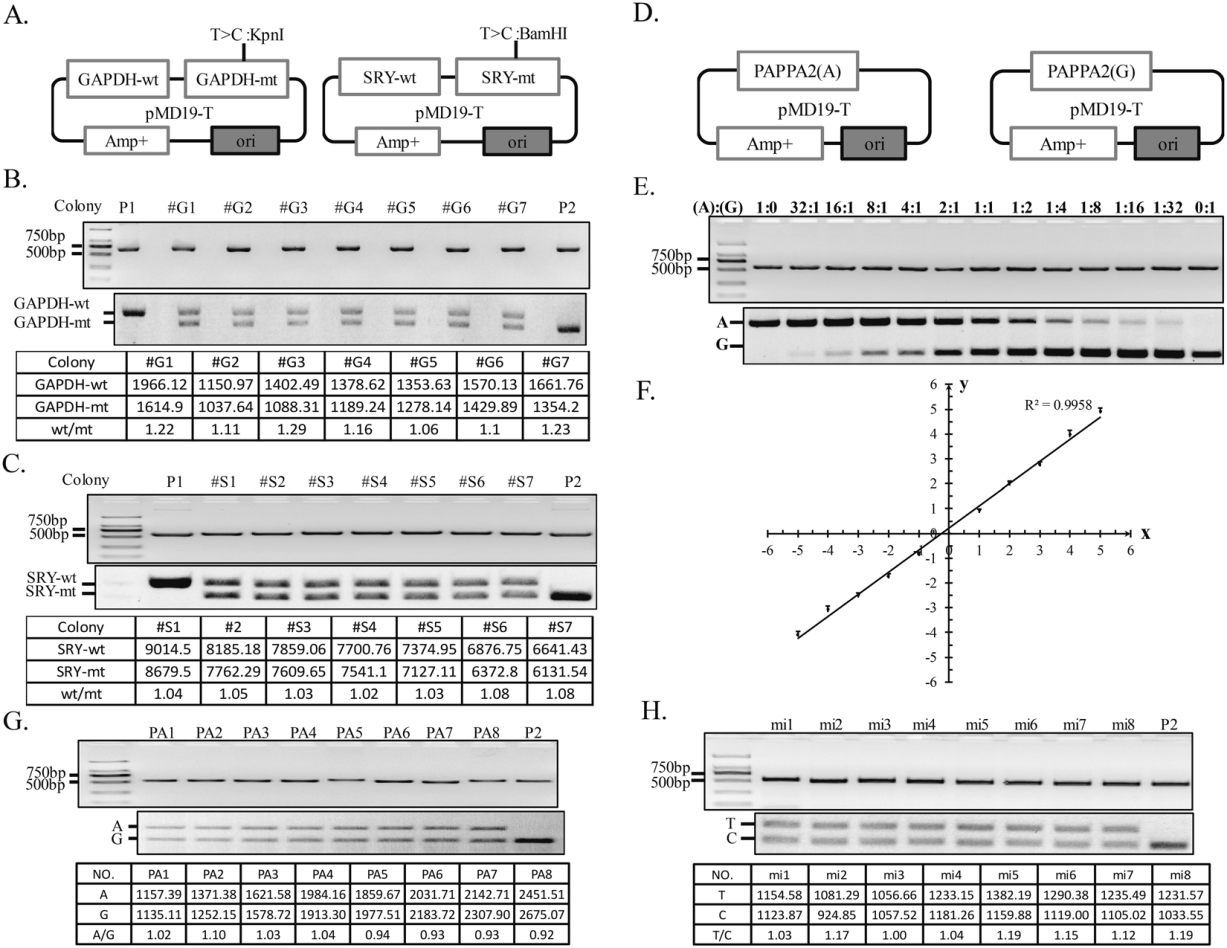
Assessment of the accuracy of fPCR-RFLP method. (**A**) The map of two vectors contain normal and mutant GAPDH or SRY gene. (**B**,**C**) The fPCR-RFLP results of GAPDH and SRY using the constructed plasmids as template. Lanes #G1~#G7 represent 7 independent colonies of GAPDH gene; lanes #S1~#S7 represent 7 independent colonies of SRY gene. (**D**) The map of vectors containing A or G alleles of the PAPPA2 gene. (**E**) The fPCR-RFLP results of PAPPA2 gene with different input ratios of A allele and G allele. (**F**) The linear range of fPCR-RFLP method analyzed based on the results of PAPPA2 gene. Error bars represent S.D. from *n* = 3 replicates. (**G**,**H**) The results of fPCR-RFLP of PAPPA2 gene and miR-155 using genomic DNA as template. Lanes PA1~PA8 represent 8 independent individuals for PAPPA2 gene; lanes mi1~mi8 represent 8 independent individuals for miR-155 gene; P2 is the positive control of restriction enzyme digestion. Full-length gels are presented in Supplementary Fig. 3.

To assess the accuracy of the fPCR-RFLP method, plasmids containing one normal and one mutant copy of *GAPDH* or *SRY* gene were constructed (Fig. 3A). Then, the DNA level of normal and mutant gene was detected using fPCR-RFLP method. The *Kpn* I and *BamH* I were used to distinguish the normal and mutant alleles. The ratios of normal/mutant allele were 1.02 to 1.08 for *SRY* gene and 1.06 to 1.23 for *GAPDH* (Fig. 3B,C). Furthermore, plasmids with A allele or G allele of *PAPPA*2 were constructed and mixed with ratios of 32:1, 16:1, 8:1, 4:1, 2:1, 1:1, 1:2, 1:4, 1:8, 1:16, and 1:32 (Fig. 3D,E). The raw and normalized fluorescence densities from three independent experiment were shown in Supplementary Table S3. The fPCR-RFLP results showed a linear relationship (*R*^*2*^ = 0.9958) across a range of 32:1 to 1:32 (Fig. 3F). The raw value was normalized using Log_2_. We next used genomic DNA as input to assess the accuracy of fPCR-RFLP. Two genes, *PAPPA2* and *miR-155*, located at the autosome were randomly selected. The DNA copy number of the two alleles of eight heterozygotes was detected using the fPCR-RFLP method. As expected, the ratio of two alleles of *PAPPA2* and *miR-155* gene was 0.92 to 1.04 and 1.00 to 1.19, respectively (Fig. 3G,H). To further validate our results, the second round of PCR products were cloned into PMD19-T vector and 96 bacteria colonies were picked randomly for sequencing. The result showed that the ratio of AA and GG genotypes was 1.05 (see Supplementary Fig. S2A, D). Therefore, we conclude that fPCR-RFLP is an efficient method for detection of ADE genes.

### Validation of ADE gene using fPCR-RFLP method

**Figure 4:**
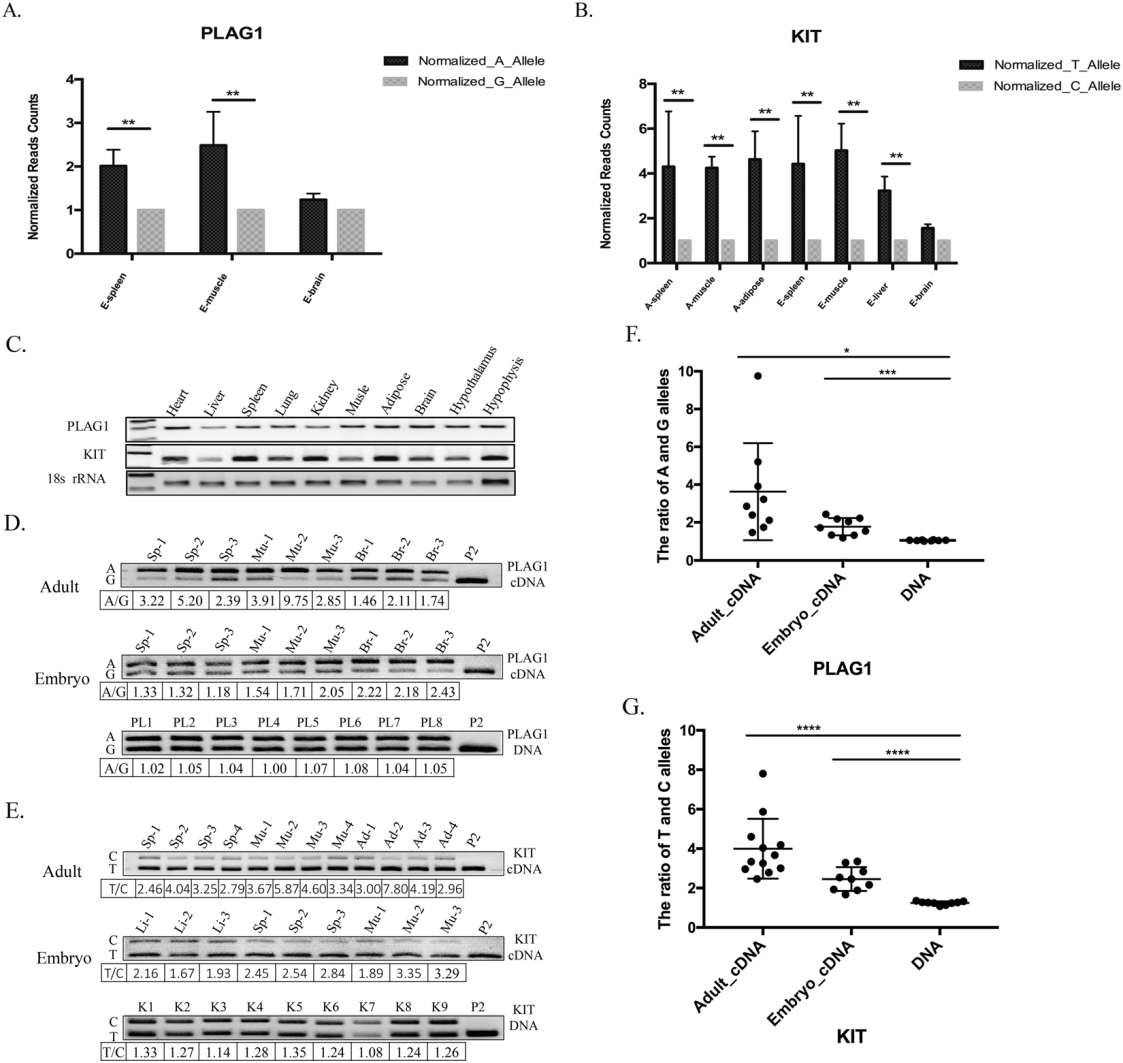
Validation of ADE of PLAG1 and KIT gene using fPCR-RFLP method. (**A**,**B**) The expression difference of two alleles of PLAG1 and KIT genes detected by RNA-seq in several pig tissues at different developmental stages. A, adult; E, embryo. (**C**) The tissue distribution of PLAG1 and KIT genes. 18 s rRNA used as internal control. (**D**,**E**) Detection of two ADE genes (PLAG1 and KIT) using fPCR-RFLP from three individuals. Sp, spleen; Mu, Muscle; Br, brain; Li, liver; Ad, adipose. Lanes PL1~PL8 represent the fPCR-RFLP results of PLAG1 gene in 8 independent individuals. Lanes K1~K9 represent the fPCR-RFLP results of KIT gene in 9 independent individuals. P2 is the positive control of restriction enzyme digestion. (**F**) The ratio of A and G allele of PLAG1 gene at cDNA and DNA level. Data were pooled from D, and t test was used to check statistics. (**G**) The ratio of T and C allele of KIT gene at cDNA and DNA level. Data were pooled from E, and t test was used to check statistics. Full-length gels are presented in Supplementary Fig. 3.

*KIT* and *PLAG1* are two allelic differentially expressed genes in pigs that were identified through RNA-Seq^42,43^. The expression of the A allele of *PLAG1* gene was significantly higher than that of G allele in spleen and muscle, and was slightly higher in brain tissue of 95-day embryo (Fig. 4A). Similarly, the expression of T allele of the *KIT* gene was significantly higher than that of the C allele in spleen, muscle, and liver, and was slightly higher in brain tissue of 95-day embryo; T allele copy was also overexpressed in adipose, muscle, and spleen tissues of 6-month-old adult pigs (Fig. 4B). We aimed to validate these ADEs using fPCR-RFLP method. Firstly, the tissue distribution of *PLAG1* and *KIT* genes was unraveled using RT-PCR. *KIT* and *PLAG1* were expressed in all 10 tissues examined (Fig. 4C). Then, the mRNA expression of the two alleles and their genomic DNA copy numbers were detected by using the fPCR-RFLP method. Tissues were collected from different individuals. The expression patterns of the two alleles of *PLAG1* gene were different in spleen, muscle, and brain, with expression level of the A allele being about 1.5-fold to 9-fold higher than that of the G allele in these tissues in adult pig (Fig. 4D, upper panel). Similarly, it was 1.3-fold to 2-fold higher in spleen, muscle, and brain tissues of 95-day embryo in three individuals, respectively (Fig. 4D, middle panel). However, the genomic DNA copy number ratio of A and G alleles was 1.00 to 1.08 in eight individuals (Fig. 4D, bottom panel). Statistically, the ratio of A and G alleles in adult and embryo cDNA level is significantly higher than DNA level, indicating ADE (Fig. 4F). For the *KIT* gene, T allele expression was about 2.5-fold to 7-fold higher than that of the C allele at mRNA level in spleen, muscle, and adipose tissues of adult pig in four individuals. It was 1.6-fold to 3-fold in liver, spleen, and muscle tissues of 95-day embryo in three individuals. The DNA copy number of T/C allele was less than 1.33-fold in nine individuals (Fig. 4E). The ratio of T and C alleles in adult and embryo cDNA level is significantly higher than DNA level, again suggesting ADE (Fig. 4G). Furthermore, we validated the results by sequencing of bacterial clones derived from PCR products and revealed a ratio of 3.87 for T/C alleles of *KIT* gene and a ratio of 1.82 for A/G alleles of *PLAG1* gene (see Supplementary Fig. S2B–D).

## Discussion

In this study, we have developed a robust method for detecting ADE. This method is simple, highly accurate, and sensitive. In this method, the quantitative analysis was performed after the PCR reaction. A previous quantitative analysis was mainly performed at early or middle stages of PCR reaction^44^. Moreover, in this method, only one pair of primers was used, which could avoid potential bias caused by amplification efficiency rendered by different primers. Particularly, a second round of PCR with only one cycle was employed to eliminate interference from the heterodimer DNA molecule. Classical RFLP methods were used to distinguish the two alleles. For the SNP sites that could not be recognized by any restriction enzyme, a non-FAM labeling mismatch primer was used to artificially create a restriction site in the amplified product to allow the following RFLP analysis^45,46^. To increase the fluorescence density, we designed an overhang labeling primers to reduce the cross fluorescence interference between two FAM. These are the major differences between our method and previous quantitative methods^44,47^.

Our method exhibits a perfectly positive linear relationship between fluorescence density and the number of DNA molecule copies. This confirms that the number of molecules could be quantified using fluorescence density, and increasing the fluorescence density of a single molecule may improve the sensitivity of this method. Therefore, we tested different types of labeling to search for increasing fluorescence density. Interestingly, fluorescence interference was observed when two FAMs had less than three nucleotides span (see Supplementary Fig. S1). Also, we found that fluorescence density was significantly increased after heat denaturation (see Supplementary Fig. S1C). A possible reason for that is that heat denaturation creates distance between two FAMs to reduce fluorescence interference. Therefore, the over-hang labeling primers were designed, and as expected, the fluorescence density was stronger than other labeling types. For over-hang labeling, the 3′ terminal of the template DNA in the second round of PCR contains at least one nucleotide mismatch with the FAM labeling primer (Fig. 1). A 5-bp gap between two FAM labeled nucleotides was recommended in this study. Our data showed that it could precisely quantify the ADE within a range of ratio of 1:32 to 32:1.

Taqman PCR and allele-specific real-time PCR are commonly used for ADE detection^48,49^. However, these methods suffer a major problem, i.e., it is difficult to distinguish two alleles on the same locus^22–24^. Besides, two pairs of primers are used for these methods, which cause bias due to different amplification efficiency. Comparatively, our method can easily distinguish two alleles based on PCR-RFLP. Also, only one pair of primer is used, which could avoid the bias of amplification by using different primers.

Further, the allelic expressions of PLAG1 and KIT were studied. They are two candidate genes associated with growth and MCH traits based on GWAS studies^42,43^. Our fPCR-RFLP results showed that the ratio of the two alleles is 1:1 at DNA level, whereas it is significantly different at mRNA level, confirming that they are ADE genes, at least in our analysed individuals. This illustrates fPCR-RLFP as a reliable method to detect ADE gene. Of note, we notice that the ADE results in the same tissue type differ in different individuals (Fig. 4D,E). This would highlight that population heterogeneity (i.e., biological variation) is likely the main cause of variation in results, but other factors that impact on gene expression and splicing may also contribute, such as the abundance of cis-acting loci^50^, DNA methylation^51^, inheritance of allele-specific epigenetic marks^52^, and different matrix^53^. Further studies are needed to investigate whether these SNPs are casual mutations located within regulatory regions and the ADE of PLAG1 and KIT are responsible for variations linked to the traits.

## Methods and Materials

### Plasmid construction

Vectors with normal and mutant copies of GAPDH or SRY gene were constructed by In-Fusion^®^ HD Cloning Kit (Clontech). The normal copy sequences were amplified by PCR using pig genomic DNA. The mutant copy sequences were generated by point mutation strategy. In-Fusion PCR primers were designed by an online tool (http://www.clontech.com/US/Products/Cloning_and_Competent_Cells/Selection_Guides/Online_In-Fusion_Tools). Recombination products were ligated to PMD19-T vector by TA cloning. Then, ligation mixture was transformed into Trans1-T1 competent cells (Transgen). The plasmids were extracted by using TaKaRa MiniBEST Plasmid Purification Kit (TaKaRa). All gene specific primers and 6-FAM labeled primers are diluted to a final concentration of 10 μM and listed in Supplementary Table S1.

### Total RNA extraction and reverse transcription

Tissues were disrupted in TransZol Up (Transgen) with a homogenizer. RNA was extracted according to the manufacturer’s instructions. cDNAs were synthesized using the PrimeScript™ RT reagent Kit with gDNA Eraser (TaKaRa). The cDNA products were used as PCR template to identify heterozygous individuals and to quantify allele expression.

### Fluorescence PCR-RFLP

The first round of PCR was performed with ∼200 ng of cDNA template and 10 μM of gene-specific forward and reverse primers in a 50 μL reaction using Premix Taq™ (Takara). PCR reaction was performed in a Veriti® 96-Well Thermal Cycler (Thermo Fisher Scientific) programmed for one cycle of 5 min at 95 °C, followed by 35 cycles of 30 s at 95 °C, 30 s at 60 °C, and 30 s at 72 °C, with a final extension step for 5 min at 72 °C. PCR products (23 μL) were used as template for the second round of PCR in a 50 μL reaction with 25 μL Premix Taq™ and 15 μM FAM-labeled primers. The PCR reaction program was set as one cycle of 5 min at 95 °C, followed by one cycle of 30 s at 95 °C, 30 s at 60 °C, and 30 s at 72 °C, with a final extension step for 5 min at 72 °C. Fluorescence PCR products (25 μL) were digested with 1 unit of the appropriate restriction enzyme in a 30 μL reaction. All digestion products were subjected to agarose gel electrophoresis with 6× loading buffer (TaKaRa) without nucleic acid dye. Finally, fluorescent image analysis was performed using Fujifilm FLA-5100 Scanner (Fujifilm). The Fluorescent density of each bands was analyzed with image analysis software Multi Gauge (Fujifilm).

### Ethics approval and consent to participate

Animals care and all the experimentation in this study were carried out in accordance with the pre-approved guidelines from the Standing Committee of Hubei People’s Congress. All experimental protocols were approved by the Ethics Committee of Huazhong Agricultural University, Wuhan City, Hubei Province, P. R. China.

## Supplementary information

Supplementary information
