# Supplementary material for: Quantification of allelic differential expression using a simple Fluorescence primer PCR-RFLP-based method
Source: Sci Rep. 2019 Apr 19;9:6334. doi: 10.1038/s41598-019-42815-5 (PMC6474871; doi:10.1038/s41598-019-42815-5)
Supplement: Supplementary file 1 — Supplementary information [file 41598_2019_42815_MOESM1_ESM.pdf]

Supplementary Information for

Quantification of allelic differential expression using a simple Fluorescence primer PCR-RFLP-based method

Changzhi Zhao[1], Shengsong Xie[1, 2], Hui Wu[1], Yu Luan[1], Suqin Hu[1], Juan Ni[1], Ruiyi Lin[1], Shuhong Zhao[1, 2], Dingxiao Zhang[1, 2*] and Xinyun Li[1, 2*]

[1]Key Laboratory of Agricultural Animal Genetics, Breeding, and Reproduction of the Ministry of Education & Key Lab of Swine Genetics and Breeding of Ministry of Agriculture and Rural Affairs, Huazhong Agricultural University, Wuhan 430070, P. R. China;

[2]The Cooperative Innovation Center for Sustainable Pig Production, Huazhong Agricultural University, Wuhan 430070, P. R. China.

Corresponding authors: Xinyun Li (xyli@mail.hzau.edu.cn), Dingxiao Zhang (Zhangdingxiao1980@yahoo.com).

This file includes:

Supplementary Figures 1-3

Supplementary Tables 1-3

Supplementary Figures

A.

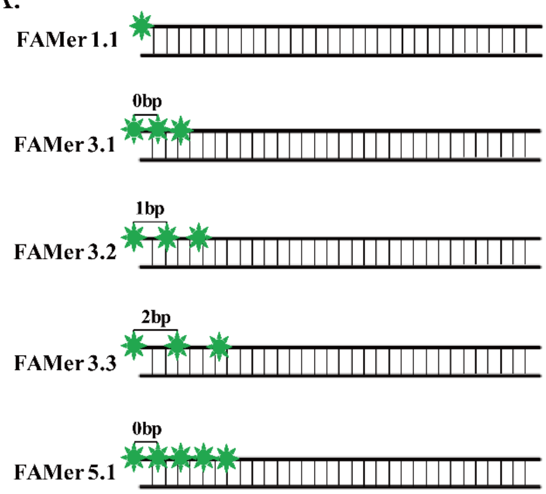

B.

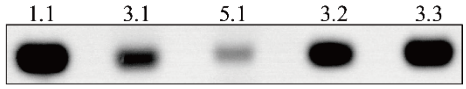

Primer	1.1	3.1	5.1	3.2	3.3
Fluorescence Density	3085.79	267.38	104.66	1292.39	1829.44

C.

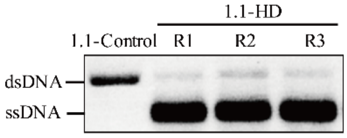

DNA types	dsDNA	ssDNA		
Primer	1.1-control	R1	R2	R3
Fluorescence Density	335.23	829.55	911.50	1074.99

Supplementary Figure 1. The position and number of the fluorophore affected the fluorescence density. **(A)** A panel of FAMers tested in (B) and (C). **(B, C)** Quantification of the fluorescence density of FAMers. Lanes R1~R3 represent three repeats. dsDNA, double strand DNA; ssDNA, single strand DNA; HD, heat denaturation. Full-length gels are presented in **Supplementary Figure 3.**

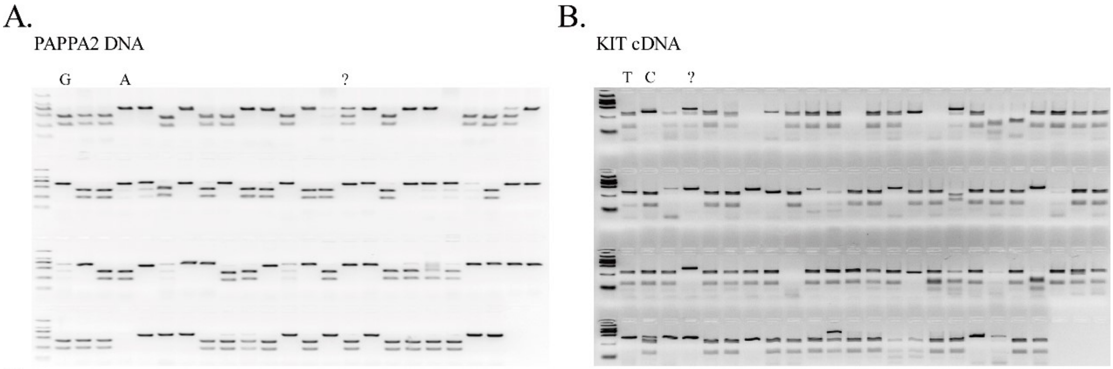

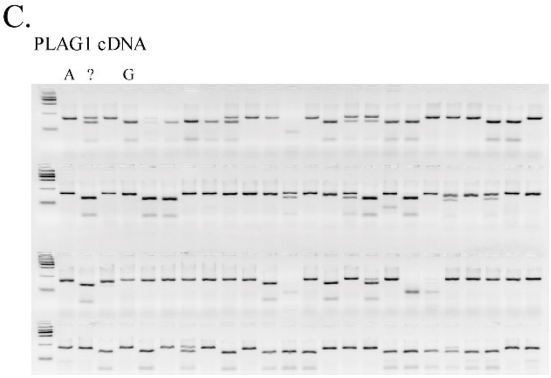

D.

Gene	Genotype		
PAPPA2 DNA (PA1)	AA (39)	GG (37)	AA:GG=1.05
KIT cDNA (Adult Ad-1)	TT (58)	CC (15)	TT:CC=3.87
PLAG1 cDNA (Br-1)	AA (51)	GG (28)	AA:GG=1.82

Supplementary Figure 2. Genotyping results. **(A-C)** The second round PCR products were cloned into PMD19-T vector; 96 bacteria colonies for each gene were picked for genotyping. ? represents the uncertain genotype. **(D)** The number of different genotypes is counted. Full-length gels are presented in **Supplementary Figure 3.**

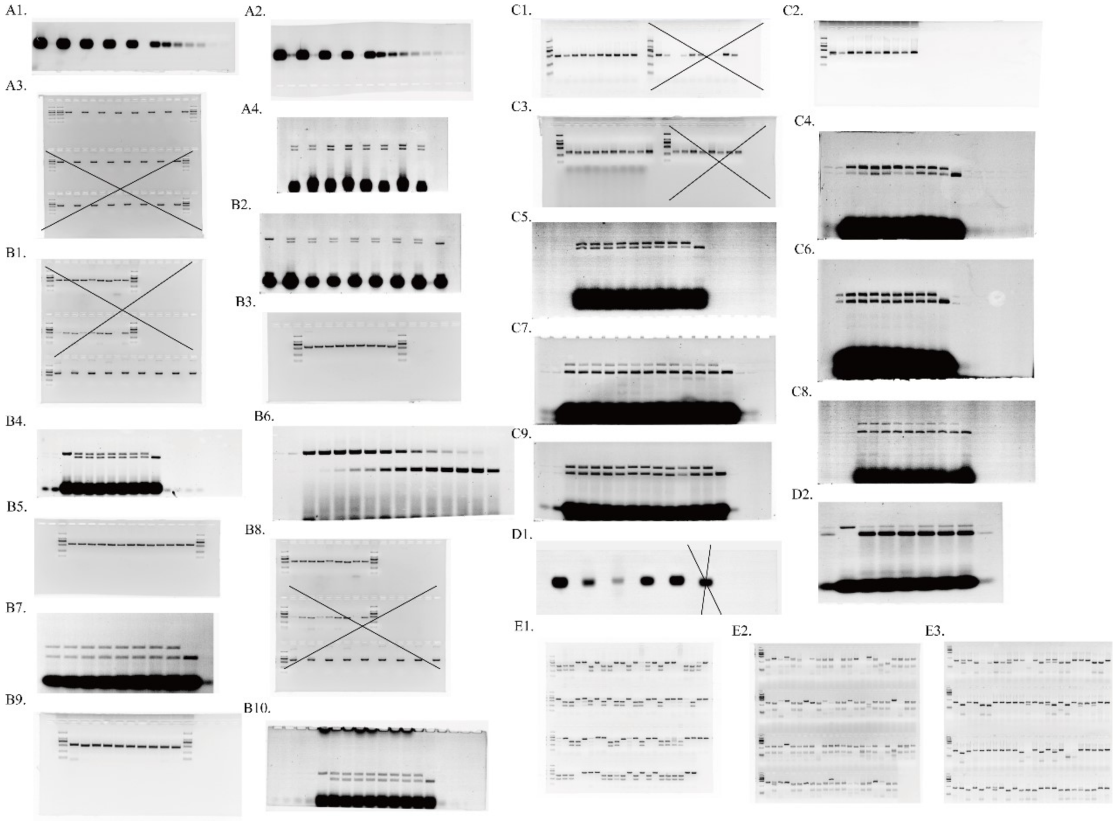

Supplementary Figure 3. The original Full-length gel images with irrelevant lanes being crossed.

A1 and A2 (associated with Figure 2A): Electrophoresis of serial diluted 1× FAMer or 2× FAMer-labeled primers, respectively.

A3 and A4 (associated with Figure 2F): fPCR-RFLP result for GAPDH using different FAMers.

B1 and B2 (associated with Figure 3B): fPCR-RFLP assay of GAPDH

with normal and mutant copies of GAPDH gene.

B3 and B4 (associated with Figure 3C): fPCR-RFLP assay of SRY with normal and mutant copies of SRY gene.

B5 and B6 (associated with Figure 3E): fPCR-RFLP assay of PAPPA2 gene with different ratios of A allele and G allele.

B7 and B8 (associated with Figure 3G): fPCR-RFLP assay of PAPPA2 gene using genomic DNA as template.

B9 and B10 (associated with Figure 3H): fPCR-RFLP assay of miR-155 using genomic DNA as template.

C1,C2, and C3 (associated with Figure 4C): Tissue expression profile of PLAG1, KIT, 18S genes.

C4,C5, and C6 (associated with Figure 4D): fPCR-RFLP result for PLAG1 gene.

C7,C8, and C9 (associated with Figure 4E): fPCR-RFLP result for KIT gene.

D1 (associated with Supplementary Figure 1B): Electrophoresis of different FAMers.

D2 (associated with Supplementary Figure 1C): Heat denaturation of FAMer.

E1 (associated with Supplementary Figure 2A): Genotyping results for PAPPA2 gene DNA.

E2 (associated with Supplementary Figure 2B): Genotyping results for

KIT gene cDNA.

E3 (associated with Supplementary Figure 2C): Genotyping results for PLAG1 gene cDNA.

Supplementary Tables

Supplementary Table 1. Primers used in this study.

**Primers**	**Sequence (5'-3')**	**Tm (℃)**	**Types of assays**
1x FAMer	**A**$_{\text{FAM}}$CTCGTCCTTCTTTCCCAGGTGT	-	FAM labeled primers
2x FAMer	**A**$_{\text{FAM}}$TCTAG**T**$_{\text{FAM}}$ ACACTCGTCCTTCTTTCCCAGGTGT	-	
FAMer 1.1	**G**$_{\text{FAM}}$GAGCCTTCCTCTTCACCTGCCGCAGATACACAGC	60	
FAMer 1.2	**A**$_{\text{FAM}}$TCTAGTACGGAGCCTTCCTCTTCACCTGCCGCAGATACACAGC	60	
FAMer 2.1	**G**$_{\text{FAM}}$GAGCC**T**$_{\text{FAM}}$TCCTCTTCACCTGCCGCAGATACACAGC	60	
FAMer 2.2	**A**$_{\text{FAM}}$TCTAG**T**$_{\text{FAM}}$ACGGAGCCTTCCTCTTCACCTGCCGCAGATACACAGC	60	
FAMer 2.3	**G**$_{\text{FAM}}$GAGCC**T**$_{\text{FAM}}$GTTCCTCTTCACCTGCCGCAGATACACAGC	60	
FAMer 2.4	**G**$_{\text{FAM}}$GAGCCTTCCTC**T**$_{\text{FAM}}$TCACCTGCCGCAGATACACAGC	60	
FAMer 2.5	**A**$_{\text{FAM}}$TCTAGCTACAT**T**$_{\text{FAM}}$ACGGAGCCTTCCTCTTCACCTGCCGCAGATACAC AGC	60	
FAMer 2.6	**G**$_{\text{FAM}}$GAGCCTTCCTG**T**$_{\text{FAM}}$GCTTCACCTGCCGCAGATACACAGC	60	
FAMer 2.7	**G**$_{\text{FAM}}$CATAGCTCTGACACTAGTGCGACAGAACAGCCCA**T**$_{\text{FAM}}$CCATC	-	
FAMer 3.1	**G**$_{\text{FAM}}$CATAGCTCTGACACTAG**T**$_{\text{FAM}}$GCGACAGAACAGCCCAG**T**$_{\text{FAM}}$CCATC	-	
FAMer 3.2	**T**$_{\text{FAM}}$**T**$_{\text{FAM}}$**T**$_{\text{FAM}}$GCATAGCTCTGACACTAGTGC	-	
FAMer 3.3	**T**$_{\text{FAM}}$A**T**$_{\text{FAM}}$C**T**$_{\text{FAM}}$GCATAGCTCTGACACTAGTGC	-	

FAMer 3.4	**T**$_{FAM}$AA**T**$_{FAM}$CC**T**$_{FAM}$GCATAGCTCTGACACTAGTGC	-	
FAMer 5.1	**T**$_{FAM}$**T**$_{FAM}$**T**$_{FAM}$**T**$_{FAM}$**T**$_{FAM}$GCATAGCTCTGACACTAGTGC	-	
GAPDH-FAM-F	**A**$_{FAM}$TCTAG**T**$_{FAM}$ACGGAGCCTTCCTCTTCACCTGCCGCAGATACACAGC	60	
SRY-FAM-F	**A**$_{FAM}$TC**T**$_{FAM}$AGTACAGCAGAGCCTTCAGCAACTCGGATTA	60	
PAPPA2-cDNA-FAM-F	**A**$_{FAM}$TCTAG**T**$_{FAM}$ACGACAGAACAGCCCAGCCATCATTGCAGGTGTGTT	60	
PAPPA2-DNA-FAM-F	**A**$_{FAM}$TCTAG**T**$_{FAM}$ACACTCGTCCTTCTTTCCCA	60	
Mir155-DNA-FAM-F	**A**$_{FAM}$TCTAG**T**$_{FAM}$ACGGTCTCCCCTCTGCGTTTTAGCATTTGG	60	
PLAG1-cDNA-FAM-R	**A**$_{FAM}$TCTAG**T**$_{FAM}$ACAAGGAAGCTGAACACCGACTCTGTAAGACT	58	
KIT-cDNA-FAM-F	**A**$_{FAM}$TCTAG**T**$_{FAM}$ACGGAGAAAGCAGAGGCCATGAATACAGG	58	
PLAG1-DNA-FAM-R	**A**$_{FAM}$TCTAG**T**$_{FAM}$ACAAGGAAGCTGAACACCGACTCTGTAAGACT	60	
KIT-DNA-FAM-R	**A**$_{FAM}$TCTAG**T**$_{FAM}$ACTGCTGCGTGAATAACGAGGTTCACTGTCAGCTGGT	60	
GAPDH-Fusion-5'-F	GTGAATTCGAGCTCGCCACAAGGGTTCGAGGAC	60	for constructing mutant copy
GAPDH-Fusion-5'-R	GGAAAATTTCTAGGTACCCATGACTCAGCTCC		
GAPDH-Fusion-3'-F	ACCTAGAAATTTTCCACAAAATGGCTCCCGGAGC	60	
GAPDH-Fusion-3'-R	GATCTCTAGAGGATCTGATGGCGACAATGTCCAC		
SRY-Fusion-5'-F	GTGAATTCGAGCTCGCTATAACATCCGCCGCCT	60	
SRY-Fusion-5'-R	TTCCACTTGGATCCCAGCCACTTGCTGATCTCTG		
SRY-Fusion-3'-F	GGGATCCAAGTGGAAAATGCTTACAGAAGCCGAA	60	
SRY-Fusion-3'-R	GATCTCTAGAGGATCCATGCTCCCCAGCACTAC		
GAPDH-t-769-F	tGGAGCCTTCCTCTTCACCTG	60	gene specific primers
GAPDH-769-R	GCCTTTAGGGTGGGGTCAAC		
SRY-t-691-F	tAGCAGAGCCTTCAGCAACTC	60	

SRY-691-R	CTTGCGACGAGGTCGGTATT		
PAPPA2-cDNA-t-654-F	tGACAGAACAGCCCAGCCATC	60	
PAPPA2-cDNA-654-R	CCCGTTGTACTGGGAGATCA		
PAPPA2-DNA-t-622-F	tACTCGTCCTTCTTTCCCAGG	60	
PAPPA2-DNA-622-R	CCACATTGTCACAAGCCGTT		
Mir155-DNA-t-478-F	tGGTCTCCCCTCTGCGTTTTA	60	
Mir155-DNA-478-R	ATGTAGGAGTCAGACGGAGGT		
PLAG1-cDNA-466 -F	AGCGCATTCTTCCGTGTCTC	58	
PLAG1-cDNA-466-t-R	tAAGGAAGCTGAACACCGACT		
PLAG1-DNA-466 -2F	AGCGCATTCTTCCGTGTCTC	60	
PLAG1-DNA-466-t-2R	tAAGGAAGCTGAACACCGACT		
KIT-cDNA-396-t-F	tGGAGAAAGCAGAGGCCATGA	58	
KIT-cDNA-396-R	ACAGCCACAACAGGTACAGC		
KIT-DNA-746-F	TCGTCGACCCTCCCTTGTAT	60	
KIT-DNA-746-t-R	tTGCTGCGTGAATAACGAGGT		
GAPDH-DNA-2139-F	CCACAAGGGTTCGAGGACTG	60	
GAPDH-DNA-2139-R	TGATGGCGACAATGTCCACT		
SRY-DNA-1610-F	CTATAACATCCGCCGCCTGG	60	
SRY-DNA-1610-R	CATGCTCCCCAGCACTACA		

Supplementary Table 2. The fluorescence density of 1× FAMer and 2× FAMer.

FAMer Concentration (μM)	x	Fluorescence Density (1× FAMer)	y (1× FAMer)	Fluorescence Density (2× FAMer)	y (2× FAMer)
160	7.32	8071837.70	6.97	8232615.51	7.10
80	6.32	4833445.28	6.23	5416601.60	6.50
40	5.32	2495675.12	5.28	2666439.55	5.48
20	4.32	1392834.82	4.44	1514827.91	4.66
10	3.32	735616.66	3.52	745795.20	3.64
2	1.00	146103.81	1.19	151841.90	1.34
1	0.00	64181.52	0.00	59812.27	0.00
1/2	-1.00	24728.80	-1.38	34421.94	-0.80
1/4	-2.00	11265.32	-2.51	14003.49	-2.09
1/8	-3.00	7298.29	-3.14	6187.63	-3.27
1/16	-4.00	1763.29	-5.19	5490.12	-3.45
1/32	-5.00	1061.31	-5.92	1661.48	-5.17
1/64	-6.00	-	-	1095.39	-5.77

x, FAMer concentration was normalized using Log2; y, fluorescence density was normalized using Log2.

Supplementary Table 3. The fluorescence density of the A and G alleles of PAPPA2.

(A):(G)	x	A1	G1	A2	G2	A3	G3	A1/G1	A2/G2	A3/G3	y1	y2	y3	S.D
32	5	44236.33	1823.50	46985.75	2325.89	47491.68	1910.20	24.26	20.20	24.86	4.91	4.90	4.65	0.14
16	4	48271.00	3800.40	49012.57	3291.90	46611.14	3851.20	12.70	14.89	12.10	3.98	3.86	4.21	0.18
8	3	50122.94	9115.40	54511.60	9165.10	49480.84	9387.40	5.50	5.95	5.27	2.77	2.66	2.89	0.12
4	2	35848.25	10968.15	34805.97	12112.34	37006.29	12015.64	3.27	2.87	3.08	2.02	1.88	1.84	0.09
2	1	30470.35	19768.07	30827.43	19659.10	31549.21	20864.75	1.54	1.57	1.51	0.93	0.86	0.97	0.06
1	0	21672.62	26851.71	21154.18	26356.79	22764.26	27256.24	0.81	0.80	0.84	0.00	0.00	0.00	0.00
1/2	-1	15574.39	34264.38	17141.00	35092.12	16003.82	35213.89	0.45	0.49	0.45	-0.83	-0.88	-0.72	0.08
1/4	-2	10811.37	44197.57	11019.91	49930.60	11589.31	51667.09	0.24	0.22	0.22	-1.72	-1.90	-1.86	0.09
1/8	-3	7410.23	51715.40	7499.97	59747.87	7521.30	52301.89	0.14	0.13	0.14	-2.49	-2.54	-2.68	0.10
1/16	-4	5152.84	53597.16	6003.24	54887.16	5326.71	55947.26	0.10	0.11	0.10	-3.07	-3.13	-2.88	0.13
1/32	-5	2515.07	52648.97	2102.35	52947.54	2445.76	53897.41	0.05	0.04	0.05	-4.08	-4.20	-4.34	0.13

x, (A):(G) was normalized using Log2; y, A/G was normalized using Log2. A, G represents the fluorescence density of A allele and G allele of PAPPA2 gene. Three replicates were performed. S.D represents standard deviation.
